# Prototyping of a Digital Life Course Care Platform for Preconception and Pregnancy Care: Pilot Feasibility and Acceptability Study

**DOI:** 10.2196/37537

**Published:** 2023-01-20

**Authors:** Melissa van der Windt, Sofie Karolina Maria van Zundert, Sam Schoenmakers, Lenie van Rossem, Régine Patricia Maria Steegers-Theunissen

**Affiliations:** 1 Department of Obstetrics and Gynecology Erasmus University Medical Center Rotterdam Netherlands; 2 Department of Clinical Chemistry Erasmus University Medical Center Rotterdam Netherlands

**Keywords:** eHealth, app, lifestyle, lifestyle care, life course care, preconception, periconception, pregnancy, health care, pilot

## Abstract

**Background:**

A healthy lifestyle plays a key role in the prevention of lifestyle-related diseases, including subfertility and pregnancy complications. Although the benefits of a healthy lifestyle are well-known, long-term adherence is limited. Moreover, memory for lifestyle-related information as well as medical information provided by the medical professional is often poor and insufficient. In order to innovate and improve health care for both the patients and health care professionals, we developed a prototype of a digital life course care platform (*Smarter Health* app), providing personalized lifestyle care trajectories integrated in medical care journeys.

**Objective:**

This pilot study aimed to evaluate the feasibility, defined as the actual app use, and the acceptability, which included patient satisfaction and appreciation, of the *Smarter Health* app.

**Methods:**

Between March 17, 2021, and September 30, 2021, pregnant women familiar with the Dutch language seeking tertiary preconception and pregnancy care were offered the app as part of standard medical care at the outpatient clinic *Healthy Pregnancy* of the Department of Obstetrics and Gynecology of the Erasmus University Medical Center. Three months after activation of the app, patients received a digital questionnaire consisting of aspects of feasibility and acceptability.

**Results:**

During this pilot study, 440 patients visited the outpatient clinic *Healthy Pregnancy*. Of the 440 patients, 293 (66.6%) activated the app. Of the 293 patients who activated the app, 125 (42.7%) filled out the questionnaire. Of these 125 patients, 48 (38.4%) used the app. Most app users used it occasionally and logged in 8 times during their medical care trajectory. Overall, app users were satisfied with the app (median 5-point Likert scale=2.4, IQR 2.0-3.3).

**Conclusions:**

Our findings showed that the *Smarter Health* app, which integrates lifestyle care in medical care, is a feasible health care innovation, and that patients were satisfied with the app. Follow-up and evaluation of pregnancy outcomes should be performed to further substantiate wider clinical implementation.

## Introduction

A healthy lifestyle can prevent a various range of diseases and is relevant throughout the entire life course. However, especially the periconception period is a crucial “time window” in life in which a healthy lifestyle is associated with fewer reproductive problems and fewer pregnancy complications, such as hypertensive disorders, gestational diabetes mellitus, and small-for-gestational-age newborns [1]. Moreover, detrimental periconception exposures create an unfavorable environment for the preconception maturing oocytes and the postconception developing embryo and placenta, and subsequently cause transgenerational health effects [2]. Thus, it is crucial to invest in the adoption of a healthy lifestyle for prepregnant and pregnant women considering its short- and long-term health effects.

Changing lifestyle behaviors is difficult, and maintaining lifestyle improvements in the long term is even more challenging. Nevertheless, during the periconception period and in early parenthood, couples are more motivated to adopt a healthy lifestyle [3]. In 2018, the Erasmus University Medical Center implemented and evaluated “blended” periconception lifestyle care, combining a face-to-face counselling session at the outpatient clinic *Healthy Pregnancy* of the Department of Obstetrics and Gynecology, with the 26-week eHealth coaching program “Smarter Pregnancy” [4-6]. This health care service is a proven, effective method to increase healthy lifestyle behaviors among prepregnant and pregnant women and their partners.

**Figure 1:**
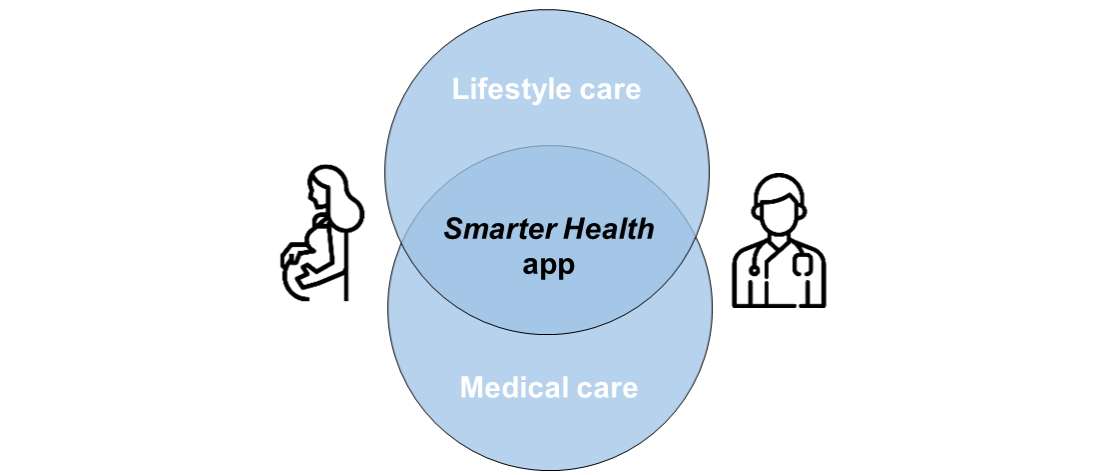
Digital life course care platform combining lifestyle care with medical care, which enhances the exchange of information between the patient and health care provider and supports health care delivery.

It is our ambition to further innovate and improve health care by implementing a digital life course care platform (*Smarter Health* app) providing lifestyle care integrated in medical care (Figure 1). This app can support health care professionals by coaching women who are contemplating pregnancy or are already pregnant toward a healthy lifestyle, but also benefits the user by increasing self-management and information access. Patient portals have shown to improve patient safety and communication, but they do not have an impact on health outcomes, whereas existing evidence-based eHealth apps for lifestyle coaching have shown to improve health outcomes [7]. Therefore, we expect that integrating patient portals with evidence-based lifestyle care trajectories will perform better on health outcomes than patient portals or e-lifestyle coaching alone.

By integrating lifestyle care in medical care journeys, the patient experiences a personalized digital journey during which information is offered when it is most relevant for the patient to receive. We expect that such an app results in better-prepared patients for their appointments with the health care provider, which enhances exchange of information. Furthermore, the patient is supported by personalized coaching and an informative system that is continuously available and not restricted to the short and relatively limited appointments with the health care professional. Herewith, patients self-manage their care pathway to improve their lifestyle, which may result in less pregnancy complications. In addition, the relatively limited appointments with health care professionals can be made more tailored. Here, we evaluated the prototype of the *Smarter Health* app on feasibility and acceptability. The results will provide information to further improve this health care innovation, and ultimately, will support wider implementation.

## Methods

### Participants and Recruitment

Eligible patients for this pilot study included women contemplating pregnancy or pregnant women familiar with the Dutch language seeking tertiary care and visiting the outpatient clinic *Healthy Pregnancy* of the Department of Obstetrics and Gynecology of the Erasmus University Medical Center [5]. All eligible patients were offered lifestyle care integrated in the *Smarter Health* app as part of standard medical care at the outpatient clinic *Healthy Pregnancy*. This pilot study covered a 6-month period, from March 17, 2021, to September 30, 2021. To evaluate the feasibility and acceptability of the *Smarter Health* app, we focused on pregnant patients since these patients had several hospital appointments in this time frame.

### Ethics Approval

The study was conducted in accordance with the principles laid down in the Declaration of Helsinki and Good Clinical Practice. Prior to the study, ethical clearance was sought from the Medical Ethics Committee Erasmus University Medical Center (MEC-2020-0797). This Committee stated that the Medical Research Involving Human Subjects Act does not apply to this study.

### Implementation of Health Care Innovation

The content of the *Smarter Health* app has been developed for preconception and pregnancy care and is displayed on the platform MediMapp of Soulve Innovations, Utrecht, the Netherlands, a professional IT company specialized in building eHealth apps. Before the *Smarter Health* app was implemented into standard care, the content and the interface of the app were discussed in detail with health care professionals, including gynecologists, nurses, outpatient clinic assistants and counselors of the outpatient clinic *Healthy Pregnancy*, and women of reproductive age. Comments and suggestions were processed by Soulve Innovations, resulting in the *Smarter Health* app in its current form. A few days prior to their appointment at the outpatient clinic *Healthy Pregnancy*, patients received a letter with instructions on how to activate and download the *Smarter Health* app. During the face-to-face consultation at *Healthy Pregnancy*, the counselor, a trained medical doctor, activated the app together with the patient.

**Figure 2:**
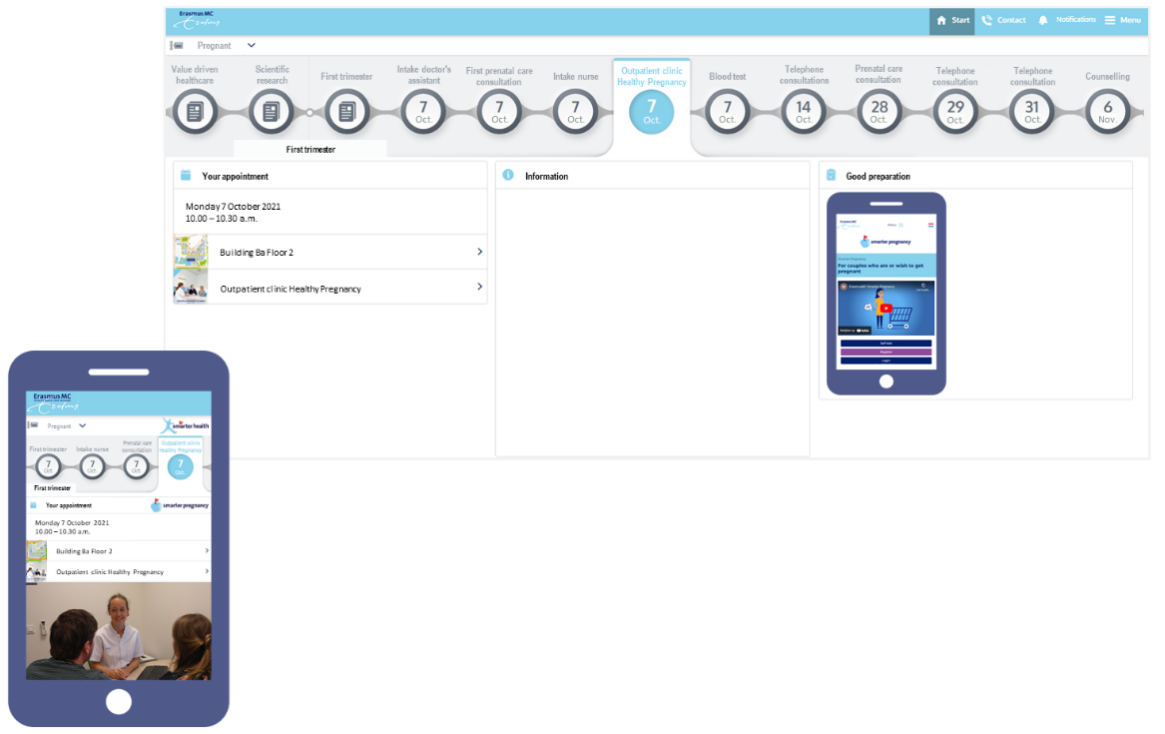
Interface of the Smarter Health app.

The interface of the *Smarter Health* app is illustrated in Figure 2. The *Smarter Health* app comprises a personalized digital patient journey, which provides information on lifestyle and relevant medical issues at the time it is most relevant. For example, information about a glucose day curve is provided to women diagnosed with gestational diabetes. This is a stand-alone app including links to reliable informative sources and evidence-based stand-alone lifestyle coaching programs, such as the English [6] and Dutch [8] versions of *Smarter Pregnancy* [5,9,10]. Patients have the option to download and share their individual results from this and other lifestyle coaching programs with their health care providers or others [5]. However, the *Smarter Health* app itself cannot be used to send data to health care providers. Through push notifications and in-app messages between the regular hospital checkups, the flow of information was spread throughout pregnancy. Since the information was part of standard care, it could potentially be found via other channels, though less structured and less well-timed.

### Data Collection

Baseline characteristics such as age, ethnicity, and parity were retrieved from medical records. The weight and height of the patients were measured by the counselor during the face-to-face consultation at the outpatient clinic *Healthy Pregnancy*. BMI was calculated by dividing the patient’s weight in kilograms by the square of the patient’s height in meters (kg/m^2^) [11].

Three months after activation of the *Smarter Health* app, the patients received a digital questionnaire consisting of 14 statements about the app, including aspects of feasibility and acceptability. This questionnaire was a short, more “doable” version of previously published validated questionnaires used in eHealth research and evaluation of the implementation of apps from the patient’s point of view [12-15]. Patients were asked to specify their level of agreement to a statement using 5-point Likert scale ranging from 1 to 5, with 1 being “strongly agree” and 5 being “strongly disagree.” In accordance with the study of Overdijkink et all [7], we referred to feasibility from a patients’ point of view and defined it as the actual app use, including the activation rate, self-reported app use, and the frequency of using the app retrieved from the app insights. The app use also provided insight into the feasibility of implementing the *Smarter Health* app in clinical practice. We assessed acceptability by self-reported user satisfaction and appreciation [7]. Statements regarding acceptability were only applicable to and answered by app users. For non–app users, an open question was included instead that covered the reasons for not using the *Smarter Health* app. Patients were reminded via email twice to fill out the questionnaire if they had not filled it out 10 and 20 days after the first email. When patients had not filled out the questionnaire after 1 month, they were reminded by telephone once to do so. Besides the reminder, no additional information was provided in the email or via telephone. Most patients reminded by telephone mentioned that they had not filled out the questionnaire yet due to a lack of time. We had no reasons to assume that these patients were less satisfied with the app than the ones who had already filled out the questionnaire.

### Statistical Analysis

Baseline characteristics of the total study population and stratified by app use (app users versus non–app users) were studied. Continuous variables were presented as mean with an SD, and categorical variables as number of individuals and percentages. The differences in baseline characteristics between respondents who stated that they used the *Smarter Health* app during their medical care trajectory and those who did not were tested using an independent 2-tailed sample *t* test for continuous variables, and the chi-squared test for categorical variables. A *P* value of ≤.05 was considered statistically significant.

To obtain the activation rate (as an aspect of feasibility), the number of patients who activated the *Smarter Health* app was divided by the total number of patients who received an invitation for the app. Since the activation rate was strongly counselor dependent, a more accurate measure of feasibility was calculated by dividing the number of respondents who reported that they used the *Smarter Health* app by the total number of respondents.

Data on the level of agreement to each statement were presented as the number of individuals and the percentages that agreed with the statement. In addition, the median 5-point Likert scale of all 14 statements combined with an IQR was calculated. All analyses were carried out using SPSS 25 (IBM Corp).

## Results

### Study Population

**Figure 3:**
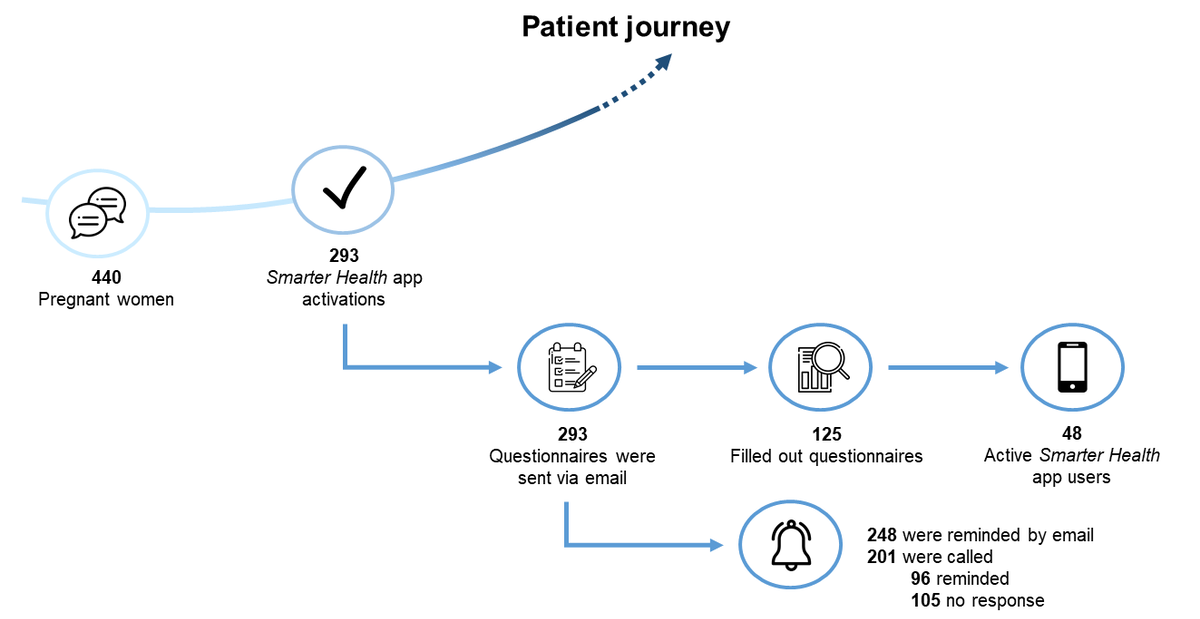
Flow diagram of participants through the phases of the study.

The participants’ flow diagram is depicted in Figure 3. From March 17, 2021, to September 30, 2021, 440 pregnant women visited the outpatient clinic *Healthy Pregnancy* of the Department of Obstetrics and Gynecology of the Erasmus University Medical Center and were eligible for participation. During this consultation, 66.6% (293/440) of the patients activated the *Smarter Health* app with the help of the counselor. In total, 42.7% (125/293) of the patients who activated the *Smarter Health* app filled out the questionnaire, and 38.4% (48/125) reported that they used the app during their medical care trajectory.

### Baseline Characteristics

Table 1 shows the baseline characteristics of the total study population stratified by app use (app users versus non–app users). In the total study population, the mean age at the moment of the consultation at the outpatient clinic *Healthy Pregnancy* was 32.6 (SD 4.0) years. The mean BMI was 26.0 (SD 6.0) kg/m^2^; 26.4% (33/125) were overweight (BMI 25-30 kg/m^2^), and 30.4% (38/125) were obese (BMI ≥30 kg/m^2^). The majority of women were of Western geographical origin (81/125, 64.8%) and multiparous (79/125, 63.2%). Age did not differ between app users and non–app users (32.4, SD 4.4 years, versus 32.8, SD 3.8 years; *P*=.59). In the subgroup of women with obesity, significantly less women were app users (13/48, 27% versus 25/77, 33%, *P*<.001). Although nonsignificant, non–app users were more frequently multiparous (54/77, 70.1% versus 25/48, 52.1%; *P*=.06), and of Western geographical origins (52/77, 67.5% versus 29/48, 60.4%, *P*=.45), compared with app users.

**Table 1:** Baseline characteristics of the total study population and stratified by app use^a^.

Baseline characteristics		Total study population (n=125)	App users (n=48)	Non–app users (n=77)	*P* value	
Age (years), mean (SD)		32.6 (4.0)	32.4 (4.4)	32.8 (3.8)	.59	
BMI (kg/m^2^), mean (SD)		26.2 (6.0)	25.5 (5.2)	26.4 (6.4)	.48	
**BMI (kg/m^2^), n (%)**						
	Overweight	33 (26.4)	12 (25.0)	21 (27.3)	.35	
	Obese	38 (30.4)	13 (27.1)	25 (32.5)	<.001	
**Parity, n (%)**						.06
	Nulliparous	46 (36.8)	23 (47.9)	23 (29.9)		
	Multiparous	79 (63.2)	25 (52.1)	54 (70.1)		
**Geographical background, n (%)**						.45
	Non-Western	44 (35.2)	19 (39.6)	25 (32.5)		
	Western	81 (64.8)	29 (60.4)	52 (67.5)		

^a^Differences were tested using an independent sample *t* test for continuous variables and using a chi-squared test for categorical variables. *P≤*.05 was considered statistically significant.

### Evaluation Outcome

#### Feasibility

**Figure 4:**
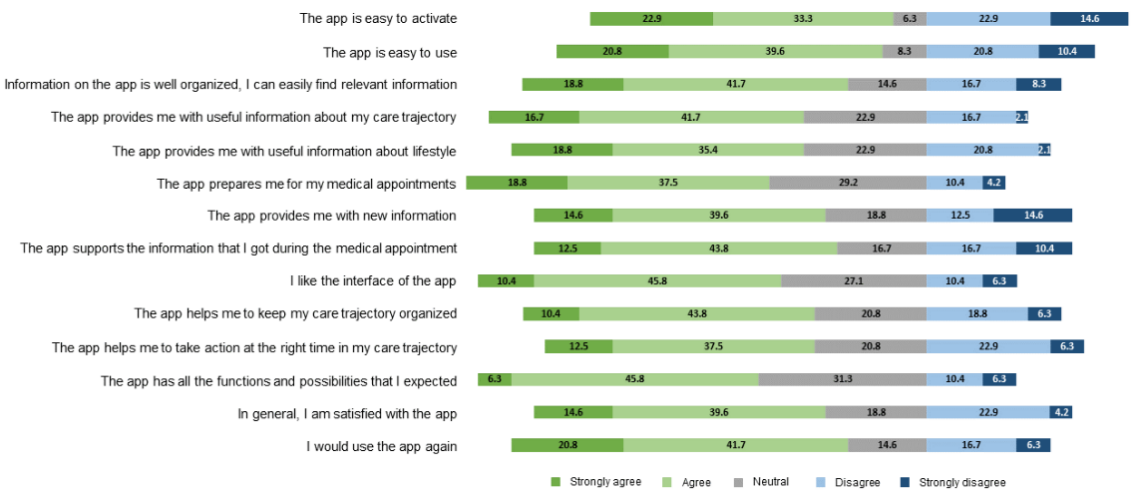
Summary of the app users’ answers on the questionnaire regarding the Smarter Health app.

As mentioned, the activation rate of the *Smarter Health* app was 66.6% (293/440), and 38.4% (48/125) of the patients who activated the app reported that they used it during their medical care trajectory. Figure 4 provides a summary of the app users’ answers on the questionnaire, illustrating their perceptions and beliefs regarding the *Smarter Health* app. The majority of the respondents who used the app (27/48, 56.2%) agreed with the statement that it was easy to activate, and 60.4% (29/48) reported that the *Smarter Health* app was easy to use. Most app users (28/48, 60.9%) used it occasionally, some (12/48, 26.1%) weekly, and a few (6/48, 13.0%) monthly. Information on app use from the app insights showed that app users logged in, on average, 8 times during their medical care trajectory.

In the response to the question, “Why have you not used the ‘Smarter Health’ app?”, a range of responses was elicited (63/77, 82% of non–app users), which could be clustered and divided into categories described here. Almost half of the non–app users (31/77, 49.2%) stated that they did not need the information in the *Smarter Health* app since they were aware of the general recommendations or already had a “healthy lifestyle.” Other commonly mentioned reasons by non–app users were a lack of time (10/77, 15.9%), difficulties with logging in (8/77, 12.7%), and an unmanageable amount of information (4/77, 6.7%). Moreover, 7 non–app users (11.1%) admitted to have forgotten about the *Smarter Health* app. In 2 (3.2%) cases, the patients switched to another hospital shortly after their first visit at the outpatient of the Department of Obstetrics and Gynecology of the Erasmus University Medical Center. Finally, 1 patient (1.7%) with a non-Western background stated that the level of the Dutch language was too difficult for her.

#### Acceptability

It is apparent from Figure 4 that almost two-thirds of the app users agreed with each statement (median 5-point Likert scale=2.4, IQR 2.0-3.3). The majority of the app users (29/48, 61%) agreed with the statement that information in the *Smarter Health* app was well organized and could be easily found. Almost 60% of the app users (27/48, 56%) commented that they liked the interface of the *Smarter Health* app. More than half of the app users indicated that the *Smarter Health* app provided them with useful information about their medical care trajectory (28/48, 58%) and lifestyle (26/48, 54%), and prepared them for their medical appointments (26/48, 56%). Most app users reported that the information in the *Smarter Health* app was new (26/48, 54%) and supported the information provided during the medical appointment (27/48, 56%). In addition, over half of the app users thought that the *Smarter Health* app helped them to keep their medical care trajectory organized (26/48, 54%) and to take action at the right time (24/48, 50%).

When asked whether the app users were satisfied with the *Smarter Health* app, 54% (26/48) reported that they were satisfied with the app, 52% (25/48) reported that the app had all the functions and possibilities that they expected, and 63% (30/48) would use the app again.

## Discussion

### Principal Results

This pilot study evaluated the feasibility and acceptability of the *Smarter Health* app during pregnancy, which was integrated in standard care. The *Smarter Health* app showed to be a feasible health care innovation since two-thirds of the patients activated the app, and more than one-third of them used it during their care trajectory and logged in on average 8 times. In general, the app users appreciated the *Smarter Health* app and were satisfied with its organization, interface, possibilities, and with the information provided about relevant medical issues, lifestyle, and their medical appointments.

### Strengths and Limitations

The main strength of this study is the integration of lifestyle care in medical care through an eHealth app. It did not only provide patients with useful information regarding lifestyle issues, but also helped them take action at the right time during their pregnancy care trajectory. As a result, patients were better prepared for their medical appointments, which enhanced the exchange of information during their medical care trajectory. Consequently, as confirmed by previous research [7], this may improve health outcomes for both mother and child. However, future research is required to investigate whether the *Smarter Health* app improves these health outcomes.

It should be noted that the *Smarter Health* app has been developed by a professional IT company specialized in building eHealth apps. The content and the user interface of the *Smarter Health* app have been discussed in detail with professionals experienced in the implementation of eHealth apps into standard care, health care professionals, counselors of the outpatient clinic *Healthy Pregnancy*, and women of reproductive age. This resulted in a continuously available and well-organized app according to the app users.

Another strength of this paper is the successful recruitment as evidenced by an activation rate of 66.6%, of which 38.4% actually used the *Smarter Health* app throughout their medical care pathway. The recruitment process consisted of the following 2 steps: (1) prior to their first medical appointment at the outpatient clinics of the Department of Obstetrics and Gynecology of the Erasmus University Medical Center, patients received an instruction letter and (2) during the face-to-face consultation at the outpatient clinic *Healthy Pregnancy*, the counselor assisted the patient with activating the *Smarter Health* app.

The most important limitation to address is the fact that the *Smarter Health* app was only available in Dutch and was not designed for illiterate patients. Since these vulnerable patients may benefit even more from this app, we recommend the further development of the *Smarter Health* app for patients who are not familiar with the Dutch language and for illiterate patients. Furthermore, due to financial and time restrictions, the evaluation of the *Smarter Health* app could not focus on the entire life course but was restricted to the duration of pregnancy. Pregnancy was chosen as time span because of its vulnerability and high plasticity [16]. Pregnancy is considered as a “window of opportunity” in which parental exposures, such as an unhealthy lifestyle, may affect developmental processes, with potential adverse outcomes in early and later life [2]. Even though we are convinced that lifestyle care should be provided throughout the entire life course, this pilot study is a step in the right direction. Another limitation is that the pragmatic setup of this study could not avoid recall and social-desirability bias completely. We have tried to minimize this by mentioning clearly in the questionnaire that their answers would be anonymized and that we were very interested in their opinion, as followed up by sending the questionnaires 3 months after activating the *Smarter Health* app, when most patients were still receiving obstetrical care in the Erasmus University Medical Center. However, some patients were not in their medical care trajectory anymore and relied on their “remembering self” rather than on their more accurate “experiencing self” when filling out the questionnaire [17]. In future investigations, it might be possible to use a different approach in which the “experiencing self” and “remembering self” are taken into account by sending questionnaires at different time points during their care trajectory and after. Additionally, when this research will be repeated on a larger scale, we recommend including questions about patient characteristics, such as socioeconomic, financial, educational, and occupational status, to investigate their association with the acceptability of the *Smarter Health* app. Finally, we have used the original (descending) order of the Likert scale including 5 points to create an interval scale, which might have influenced the results by left-side selection bias [18,19]. However, this is not supported by all studies investigating the order of scales used in self-administered surveys, which implies limited bias [20]. Moreover, since our results were consistently positive, we think that using an ascending Likert scale would not have changed our conclusions substantially.

### Comparison With Prior Work and Implications

It is proven beyond doubt that memory for medical information is often poor and inaccurate as 40%-80% of medical information provided by health care practitioners is forgotten immediately [21]. However, memory for medical information is essential for satisfaction with medical care and good adherence to recommended treatment and advice [21]. Written information is better remembered and can be used to support the spoken information given during medical appointments, which contributes to higher satisfaction rates [22]. In this study, a regular medical visit with spoken information is combined with a continually available app with written information. This valuable combination could explain the high satisfaction rates of our participants.

Since this is an emerging field in both research and practice, only a few comparable smartphone apps with a digital care pathway for specific patient groups have been designed and tested. As an illustration, an app providing a digital care pathway for patients undergoing spine surgery revealed results similar to our study, with also more than half of the patients saying it was helpful in preparing for their medical procedure. In addition, most of them (96%) reported that they would recommend the app to a friend or family member [23].

Besides evaluating feasibility and acceptability, evidence on the efficacy of the *Smarter Health* app should further substantiate its implementation in practice. Another future implication is to extend the features of the *Smarter Health* app with the capability of including data obtained by wearables. This could enable the collection of important patient data and remote monitoring, which could further increase self-management, personalized medicine, and patient satisfaction [24].

### Conclusions

This pilot study showed that the prototype of the *Smarter Health* app is a feasible and usable health care innovation during pregnancy. Using lifestyle care as a road map for medical care pathways in an app enhanced the exchange of information between health care professionals and patients, and increased patients’ self-management and information access. The next step is to expand the *Smarter Health* app by developing personalized, life course–phased, and context-specific medical pathways and lifestyle coaching programs for all medical disciplines. Despite the promising results, future research will need to be undertaken to investigate the possible health benefits of the *Smarter Health* app for both mother and child.
